# Vardenafil and Tamsulosin in the Management of Ureteral Stent–Related Symptoms: A Prospective Comparative Study

**DOI:** 10.5152/tud.2025.24111

**Published:** 2025-07-29

**Authors:** Ahmed Abdellatif, Ahmed Mohamed, Amr Massoud, Ahmed Abd Elbary, Akram Elmarakbie

**Affiliations:** 1 Department of Urology, Beni-Suef University Faculty of Medicine, Beni Suef, Egypt; 2 Department of Urology, Beni Suef University Hospital, Beni Suef, Egypt

**Keywords:** Tamsulosin, ureteral stent–related symptoms, USSQ, vardenafil

## Abstract

**Objective:**

This study aimed to compare the effectiveness of vardenafil, a phosphodiesterase-5 inhibitor, and tamsulosin, an alpha-blocker, in the management of ureteral srtent–elated symptoms.

**Methods:**

A total of 208 patients who underwent ureteric stent placement after the removal of ureteric stones were enrolled and randomly divided to receive either vardenafil 10 mg daily or tamsulosin 0.4 mg daily for a duration of 3 weeks. The validated Ureteral Stent Symptom Questionnaire (USSQ) was used to assess patients at baseline and after 3 weeks from starting the medications.

**Results:**

This study compared tamsulosin and vardenafil in 208 patients (101 vs. 107) with a mean age of 45.07 ± 9.5 years, predominantly male (67.4%); both groups were similar in baseline characteristics (*P* >.05). A notable statistical significant reduction in total scores from the first visit to the fourth visit (vardenafil: 136.03 to 85.01; tamsulosin: 129.9 to 97). Vardenafil showed a statistically significant improvement (*P* <.001) compared to tamsulosin across all USSQ domains except body pain, which has statistically significant improvement in the tamsulosin group. During the follow-up visits, vardenafil had statistically significant improvement in all USSQ domains; however, tamsulosin had statistically significant improvement in all USSQ domains except in sexual health (*P* = .5). Side effects were mild, with retrograde ejaculation and hypotension in the tamsulosin group and headaches in the vardenafil group.

**Conclusion:**

Vardenafil showed promising results in controlling stent-related symptoms and can be considered an alternative or adjunct medication to tamsulosin in the management of stent-related symptoms; however, this needs further exploration in larger, multi-center studies to validate these findings and optimize patient outcomes in clinical practice.

**Main Points**

- Vardenafil was effective in decreasing stent-related symptoms, with significantly improved urinary symptoms, sexual health, and work performance scores compared to baseline.
- Tamsulosin was also effective, showing significant improvement in body pain and urinary symptoms but had limited impact on sexual health and work performance, with no significant change in the sexual health score.
- Vardenafil was found to be superior in managing sexual health–related symptoms and in the work performance domain.
- Both medications were generally well-tolerated. In the tamsulosin group, common side effects included retrograde ejaculation and mild postural hypotension. In the vardenafil group, mild headache was the most reported side effect.

## Introduction

Double J Ureteral stents (DJ) placement is a commonly used procedure in the management of various urological conditions, including ureteral stones, strictures, and obstructions.^1^ Since its first development by Finney and Hepperlen, the DJ stents have undergone many modifications to reduce complications such as expulsion and migration.^2^ Despite these modifications and the clinical benefits of DJ stents, using them can lead to significant adverse symptoms collectively referred to as stent-related symptoms (SRS).^3^ It has been reported that these SRS can be present in approximately 80% of DJ stent cases.^4^ Double J SRS covers a wide range of symptoms, including sexual dysfunction and lower urinary tract symptoms (LUTS), as well as other symptoms. They can also lead to decreased work capacity and quality of life.^5^^,^^6^

SRS pathophysiology is not well understood, and the exact mechanism causing them remains unclear. Various theories suggested that the stent’s position, length, and materials could be associated with trigonal and ureteric irritation. This irritation causes disruptions in ureteral peristalsis, inflammation of the bladder mucosa, detrusor spasms, and urine backflow into the kidney.^7^ Bladder trigone irritation by the stent’s distal end could lead to irritative voiding symptoms particularly when the stent crosses the midline. Flank pain is most probably related to urine reflux and elevated intrapelvic pressure. One of the causes of urinary incontinence is trigonal irritation from the stent or stent migration into the urethra bypassing the sphincter. Hematuria could result directly from the procedure of inserting the stent or the surgical management of the primary pathology.^8^^,^^9^ To evaluate the impact and severity of SRS on quality of life, the Ureteral Stent Symptom Questionnaire (USSQ) was developed by Joshi et al.^10^

Advances in understanding the pathophysiology of SRS have led to continuous improvements in treatment options. An important measure in relieving the associated symptoms is enhancing ureteral stents design and materials.^11^ Additionally, SRS are commonly treated pharmacologically with alpha-blockers and antimuscarinics. Numerous studies have explored the role of these agents in managing SRS.^12^^-14^ Phosphodiesterase-5 inhibitors (PDE-5 inhibitors) are also used in the management of this condition.^15^ Vardenafil is a PDE-5 inhibitor mainly used to treat erectile dysfunction (ED).^16^ However, to the authors’ knowledge, the utilization of vardenafil in treating ureteral stent–related symptoms has not been explored before. The aim of this study was to evaluate the safety and efficacy of vardenafil in the treatment of SRS and to compare its efficacy and safety to the commonly used tamsulosin.

## Material and Methods

This was a prospective parallel comparative study conducted from November 2020 to November 2023 in the Urology department at Beni Suef University Hospital.

### Study Participants

All patients who underwent ureteric stent placement for uncomplicated ureteric stone after unilateral ureteroscopic lithotripsy (URSL) or Percutaneous nephrolithotomy (PCNL) who experienced stent-related symptoms were included in this study. A comprehensive urological, sexual, medications,and past medical history were obtained from each patient. Exclusion criteria included patients with bilateral ureteral pathology, symptomatizing benign prostatic hyperplasia, suspected prostate cancer with prostate-Specific Antigen (PSA) above 2.5 or abnormal digital rectal examination, those with a history of previous pelvic or spinal cord trauma, pregnancy, history of diagnosed ED, female sexual dysfunction, or self-reported sexual difficulties, contraindications to vardenafil or tamsulosin, concomitant urinary tract infection (UTI), patients taking antidepressants and anticholinergics, and major complications during or after the operation (migration, avulsion, perforation, sepsis, or severe intraoperative pain).

### Ethical Considerations

All study participants were provided with detailed information about the study procedures and were informed of their right to decline participation or withdraw from the study without providing any justification. Participants were assured of their privacy and all submitted information was handled with confidentiality, and written informed consent was obtained from the participants. The required administrative regulations were fulfilled. As for the ethical approval, it was obtained from the Faculty of Medicine, Beni Suef University Research Ethical Committee before commencing the work with approval number: FMBSUREC/09022020/Mohammed and date: 9th of February, 2020.

### Perioperative Care

Before the operation, history and examination, especially digital rectal examination for male patients above 50 years old were conducted. All patients received systematic examination of urine analysis, urine culture and sensitivity, serum PSA for males above 50 years, routine blood tests and radiological investigations including KUB (kidney, ureter and bladder), pelvic abdominal ultrasound and urinary tract computed tomography. Operations were done by the same surgical team. In addition, the same DJ material, diameter, and length (26) were used in this study to minimize variability. So poly-urethane 6F DJ ureteral stents were inserted in all patients.

After the operation, all patients received the same antibiotics (ciprofloxacin, 500 mg, twice daily) and anti-inflammatory medications (diclofenac potassium, 50 mg, as needed). On the morning of the first postoperative day, All patients had KUB imaging to confirm the placement of the DJ ureteral catheter. Residual stones were reported for all patients who had PCNL (25 patients), and none were reported for patients who had ureteroscopy. Eligible patients were educated about SRSs and given the validated Arabic version of the USSQ, originally developed by Joshi et al.^10^ This questionnaire has been verified to assess SRS, organising them into specific areas: urinary symptoms, physical discomfort, overall health, job performance, and sexual concerns.^10^ Participants were instructed to return 1 week later with the completed questionnaire.

### Randomization and Follow-Up

**Figure 1.**
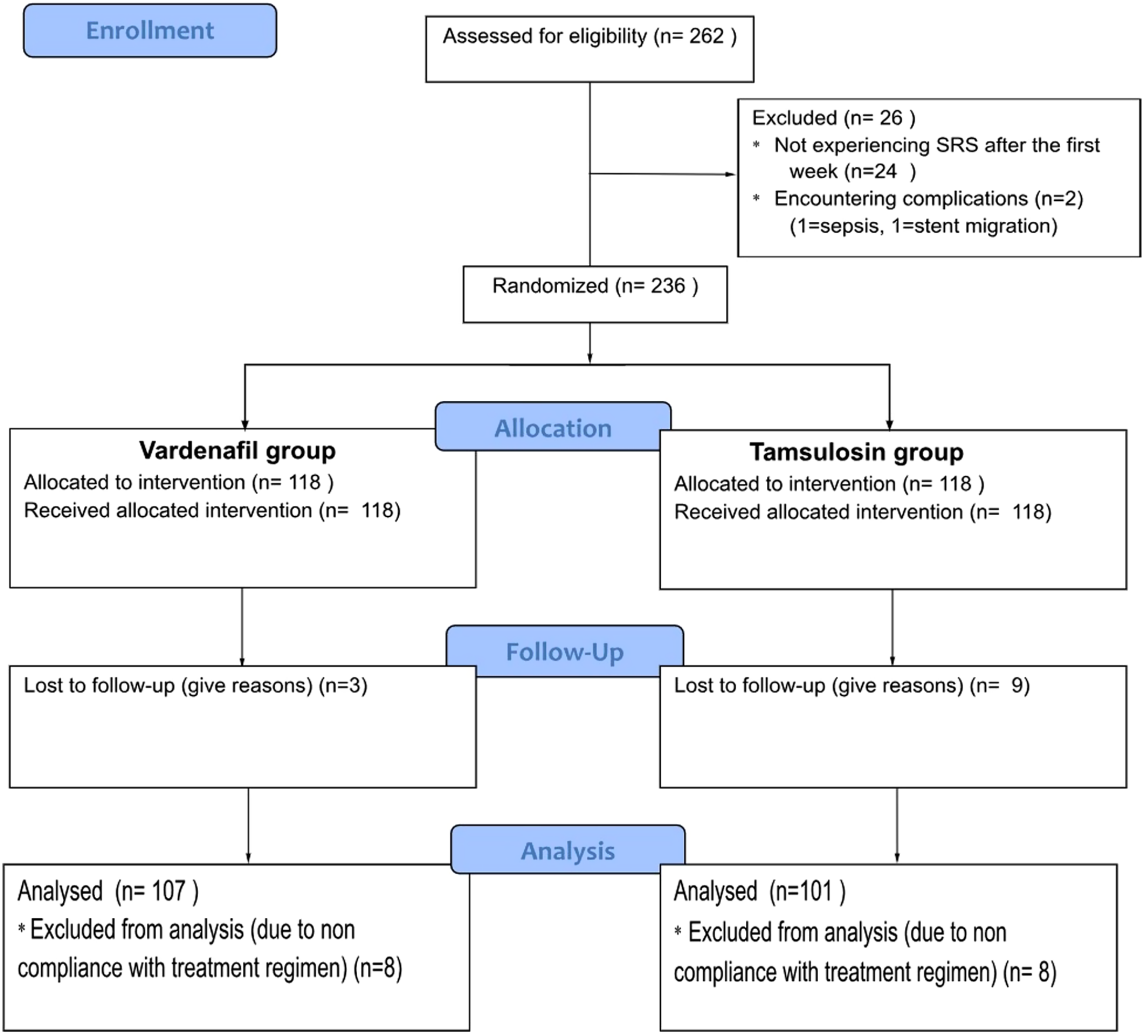
Flow diagram of the enrollment process of the participants.

The total number of patients who underwent DJ insertion was 262 patients, of whom 26 patients were excluded (2 patients experienced complications and 24 patients did not exhibit SRS). There were 4 visits after the insertion of DJ stent. The first visit was 1 week after DJ insertion (in this visit patients who did not report SRS were excluded) the second visit was 2 weeks after DJ insertion, the third visit was 3 weeks after DJ insertion and the fourth was 4 weeks after DJ insertion. The evaluation was done every visit through USSQ. A KUB was performed at each visit to verify the correct placement of the DJ stent. After 1 week of DJ stent insertion, 236 patients were randomly assigned to 2 groups using sealed envelope randomization. One group received tamsulosin 0.4 mg once daily, while the other group received vardenafil 10 mg once daily for the following 3 weeks. Patients were instructed to take the medication after the same meal each day, drink plenty of fluids throughout the day, and ensure complete bladder evacuation. In terms of follow-up, 14 patients in the vardenafil group and 17 patients in the tamsulosin arm missed follow-up. Therefore, the final analyzed number was 208 (183 patients after ureteroscopy and 25 patients after PCNL). See Figure 1.

## Results

A total of 208 patients were included in this study; 101 of them were randomized to receive tamsulosin while 107 received vardenafil. The mean age of the participants was 45.07 ± 9.5 years, and the majority of them, around 140 (67.4%) were males. Additionally, most of the patients, 146 (70.2%) were under 50 years old, while 62 (29.8%) were over 50 years. As for the vardenafil group, the majority of the participants, 69 (64.49%) were also males, with a mean age of 47.4 ± 8.6 years, while it was 40.3 ± 7.8 years for females, who composed 38 (35.51%) of the patients in the group. In the tamsulosin group, most of the patients, 71 (70.29%) were males with a mean age of 48.3 ± 9.7 years, and 30 (29.70%) patients were females with a mean age of 42 ± 10.02 years, Table 1 shows the characteristics of the included participants.

The USSQ questionnaire was used to evaluate the response to the medications in both groups. In the vardenafil group, significant effects were noted across all the domains of the questionnaire. A notable statistical significant reduction in total scores from the first visit to the fourth visit (USSQ total score: from 136.03 to 85.01; *P*-value < .001). Particularly in the urinary symptoms score, a drop by almost half of the mean score was reported, from 47.6 in the first assessment visit to 24.6 in the fourth assessment visit after starting the medication. This reduction in score was statistically significant, with a *P*-value of <.001. Sexual health score also significantly improved, from a mean of 10.3 in the first visit to 7.05 in the fourth visit, *P*-value < .001. Furthermore, participants showed an improvement in their work performance scores, with a significant decrease in the mean score from 18.03 in the first visit to 13.8 in the fourth visit of the assessment, *P*-value <.001.

Regarding the tamsulosin group, the results varied across the assessed domains. A notable statistical significant reduction in total scores from the first visit to the fourth visit (USSQ total score: from 129.9 to 97; *P*-value < .001). While some domains showed significant improvement, the mean scores for urinary symptoms and body pain dropped from 48.9 and 23.4 in the first week to 26 and 12.4 in the fourth week, respectively. The sexual health mean score also decreased from 10.3 to 9.9; however, this reduction was not statistically significant, *P*-value: .5.

Table 2 describes the comparison of the effects of tamsulosin and vardenafil on the USSQ score domains. At baseline (first visit), both groups exhibited high total USSQ scores with minimal differences between them (vardenafil: 136.03 ± 15; tamsulosin: 129.9 ± 14.7), indicating similar severity of symptoms. Over time, both groups demonstrated progressive improvements across all domains, with a notable reduction in total scores by the fourth visit (vardenafil: 85.01 ± 12; tamsulosin: 97 ± 13). The greatest improvement was observed in urinary symptoms, sexual health, and body pain, especially in the vardenafil group. Statistically significant differences emerged by the third visit and became highly significant by the fourth visit (*P* < .001), favoring vardenafil in all domains except body pain. This trend suggests that vardenafil may offer superior symptomatic relief compared to tamsulosin in patients with LUTS, particularly when evaluated over a sustained follow-up period.

Some side effects were reported by the participants during the study; however, they all continued with the study. In the tamsulosin group, 6 patients reported retrograde ejaculation, 9 patients reported mild postural hypotension, and 3 had mild headaches. Meanwhile, in the vardenafil group, 13 patients reported mild headaches, and no other side effects were stated by the participants in this group.

## Discussion

Ureteral stents play a crucial role in the endourology field; they are useful in preventing the obstruction of urinary flow in the ureter due to mucosal edema and can also facilitate the healing of the mucosa following a complicated procedure.^1^^,^^17^ Ureteral stents can dilate the ureter, potentially facilitating the passage of residual stones.^18^ A major concern that arises from the use of ureteral stents is SRS. About 32% of patients experienced sexual dysfunction, 58% reported decreased work performance, and 80% reported LUTS and pain due to the ureteral stent.^19^ Given the continuous and growing use of ureteral stents, it is crucial to address these symptoms. Several methods have been used for this purpose, which could be divided into preventive and pharmacological strategies.

Preventive strategies involve modifying stent design and using biomaterials specifically developed for this purpose, ensuring appropriate stent positioning, and adjusting stent length according to the patient’s height. Other strategies involve using stent coatings, drug-eluting stents, and providing thorough patient counselling regarding potential symptoms.^7^^,^^17^^,^^20^^,^^21^ On the other hand, It was stated that pharmacological therapy is the most effective way to control SRS. This includes alpha-blockers, anticholinergics, and analgesics.^2^^,^^3^^,^^7^^,^^22^^-^^25^ Among these alpha-blockers, tamsulosin has been extensively investigated and proven effective in alleviating stent-related symptoms. Alpha 1 adrenergic receptors specially alpha-1A and alpha-1D are found in the ureter, particularly in distal third ureter and ureterovesical junction, thus, blocking these receptors was found to be beneficial in controlling SRS by decreasing muscle contraction.^26^^,^^27^ Furthermore, the food and drug adminstration has approved PDE5 inhibitors like sildenafil, vardenafil, and tadalafil for the treatment of premature ejaculation, ED, and LUTS.^28^ Phosphodiesterase-5 receptors are present at lower ureter, bladder neck, and trigone and prostate. Antagonising these receptors could result in ureteral relaxation and facilitate stone propagation and expulsion, therefore, PDE5 inhibitors have been tried recently in treating SRS.^26^^,^^27^^,^^29^ A study showed that vardenafil was more effective and specific than sildenafil in blocking phosphodiesterase-5, and it does not affect phosphodiesterase-6, thus avoiding the risk of causing visual disturbances.^30^

When comparing tamsulosin and vardenafil in this study, notable differences were reported, across various domains of the USSQ. Compared to tamsulosin, vardenafil was found to be superior in managing sexual health-related symptoms and overall satisfaction. The most notable improvements occurred in urinary symptoms, sexual function, and bodily pain, particularly among patients taking vardenafil. By the third follow-up visit, statistically significant differences were observed, and these differences grew even more pronounced by the fourth visit, with vardenafil outperforming tamsulosin in every measured area except for body pain. These findings indicate that vardenafil may be more effective than tamsulosin in alleviating LUTS, particularly when assessed over an extended period.

Several studies investigated the efficacy of tamsulosin in reducing SRS. In a study comparing Mirabegron and tamsulosin, the latter slightly improved the urinary symptoms more than Mirabegron, however it did not affect the other domains in the USSQ.^17^ In contrast, another study found that Mirabegron was superior to tamsulosin in decreasing body and urinary symptoms, and improving the quality of life for urethral stent patients.^18^ In another study, tamsulosin was compared to tadalafil, the results showed that tadalafil was more effective in all of the assessment domains with exception of the urinary symptoms domain, in which tamsulosin was more effective.^27^ However, Aggarwal et al^26^ found that tadalafil was comparably effective to tamsulosin in decreasing urinary symptoms, and more effective in the sexual health domain, which is similar to the findings in this study. Most of these studies showed that tamsulosin is effective in reducing the urinary symptoms, this could be due to the potent impact of tamsulosin in reducing ureteric spasm, voiding pressure, and bladder outlet obstruction.^27^

Phosphodiesterase-5 inhibitors efficacy in reducing SRS have been also discussed in many studies. Two randomized trials found that tadalafil was more effective compared to placebo in decreasing body pain and urinary symptoms, and improving sexual health in stent patients.^29^^,^^31^ Improving the sexual health of patients might also be beneficial for the goal of treating stones, a randomized controlled trial found that sexual partners who were having sexual intercourse 3-4 times per week had more probability of spontaneous passage of the distal ureteral stones that are ≤ 6 mm.^32^ Additionally, sildenafil was found to be safe and effective in improving SRS.^33^ Combination therapy with tadalafil and tamsulosin was more effective and safer in relieving SRS compared to monotherapy with either of the 2 medications.^34^ Compared to a combination of solifenacin and mirabegron, tadalafil showed better results in lowering pain score, urinary symptoms, and better work activity and general condition.^35^

To the authors’ knowledge, this was the first study that assessed vardenafil’s efficacy in managing SRS. However, it has some limitations, one of the limitations of this study was the sample size, which was adequate for drawing preliminary comparisons, however future studies with larger sample sizes are necessary for the generalizability of the findings. Additionally, being a single-center study, this reduces the external validity of the results, hence more multi-centered studies are recommended. Furthermore, there was no blinding in this study, which increases the risk of bias. Future studies should also explore the mechanisms underlying the difference in efficacy that was observed between vardenafil and tamsulosin, and perform cost-effectiveness analysis. Moreover, options for combination therapies with both of these medications ought to be explored, since tamsulosin has a rapid induction of pain relief, 100% oral bioavailability and longer half-life which might enhance the efficacy of vardenafil in a combination therapy.^36^

In conclusion, this study revealed that vardenafil and tamsulosin were both effective in reducing stent-related symptoms. Vardenafil was found to be superior in managing sexual health–related symptoms and in the work performance domain. However, the findings indicated no significant difference in efficacy between the 2 medications for body pain, urinary symptoms, and general health domains. Larger sized, multi-center studies are recommended to further validate the findings of this study and explore the long-term efficacy and safety of both medications in treating stent-related symptoms.

## Figures and Tables

**Table 1.** Characteristics of the Included Participants

Item	Total (n = 208)	Vardenafil (n = 107)	Tamsulosin (n = 101)	*P*
Gender				
Males	140 (67.3)	69 (64.5)	71 (70.3)	.85
Females	68 (32.7)	38 (35.5)	30 (29.7)	
Type of operation				
Ureteroscopy patients	183 (87.98)	98 (91.6)	85 (84.2)	.69
PCNL patients	25 (12.02)	9 (8.4)	16 (15.8)	

Values are given as n (%).

PCNL, percutaneous nephrolithotomy.

**Table 2.** Comparison Between the Effect Vardenafil and Tamsulosin on Ureteral Stent Symptom Questionnaire Score Domains

Groups	USSQ Total	Urinary Symptoms	Sexual Health	Body Pain	General Health	Work Performance	Additional Problems
**First Visit**							
Vardenafil (n = 107)	136.03±15	47.6±6	10.3±2.5	24.4±4.5	24.2±3.8	18.03±3.5	11.5±2.5
Tamsulosin (n = 101)	129.9±14.7	48.9±5	10.3±2.4	23.4±4.4	25.4±3.9	13.5±3.2	8.4±2.4
*P*	.070	.125	.593	.258	.456	.278	.236
**Second Visit**							
Vardenafil (n = 107)	102.4±14	33.5±5.5	9.5±2.3	17.2±4.2	19.6±3.5	17.5±3	5.1±2.3
Tamsulosin (n = 101)	103.1±15	38.6±6	9.6±2.4	18.3±4.3	20.2±3.6	10.3±3.3	6.1±2.5
*P*	.061	.089	.191	.134	.120	.099	.151
**Third Visit**							
Vardenafil (n = 107)	73.4±13	29.2±5	8.9±2.1	15.6±4	19.2±3.3	15.8±2.8	7.9±2.1
Tamsulosin (n = 101)	96.6±14	33.9±5.5	9.3±2.3	16.3±4.2	19.7±3.5	12.2±3.1	8.1±2.3
*P*	.035	.041	.066	.048	.049	.044	.050
**Fourth Visit**							
Vardenafil (n = 107)	85.01±12	24.6±5	7.05±2	13.03±3.8	18.03±3.2	13.8±2.6	8.5±3.8
Tamsulosin (n = 101)	97±13	26.0±5	9.9±2.5	12.4±4.1	18.4±3.4	18.4±3.1	11.9±2.5
*P*	<.001	<.001	<.001	<.001	<.001	<.001	<.001

USSQ, Ureteral Stent Symptom Questionnaire. Values are given as mean±standard deviation

## Data Availability

The data that support the findings of this study are available on request from the corresponding author.
